# 111 Optimizing an Outpatient Mhealth Intervention for Dallas with Burns: A Mixed Methods Study

**DOI:** 10.1093/jbcr/irac012.114

**Published:** 2022-03-23

**Authors:** Aaron Lesher, Yulia Gavrilova

**Affiliations:** South Carolina Burn Center at MUSC, Charleston, South Carolina; Medical University of South Carolina, Charleston, South Carolina

## Abstract

**Introduction:**

Pediatric burn injury remains one of the most common traumatic injuries in childhood. Fortunately, up to 90% of pediatric burns may be treated safely in the outpatient setting after appropriate burn triage. Patients face significant geographic disparities in access to expert burn care due to the regionalization of burn care. To aid patients and their families during acute burn recovery, a smartphone app was developed to improve patient outcomes, increase access to care, and streamline communication. The purpose of this study is to evaluate patient-derived feedback to optimize this burn app prior to subsequent efficacy testing.

**Methods:**

The burn app was evaluated using a mixed-method approach consisting of qualitative semi-structured interviews and quantitative usability data gathered from caregivers of pediatric burn patients who utilized the app during the treatment phase of their child’s burn injury. Usability data were collected using a psychometrically validated mHealth App Usability Questionnaire (MAUQ). To analyze the interview transcriptions, we followed the Braun and Clark (2006) framework of thematic analysis. The research questions focused on caregiver perceptions of smartphone-enhanced pediatric burn care and potential app improvements, including acceptability, usability, technical preferences, and emotional perceptions.

**Results:**

14 caregivers (93% women; M age = 36) completed the study. Overall MAUQ scores (M = 6.46; SD = .62) indicated high app usability. Ease of Use & Satisfaction (M = 6.66; SD = .42), System Information Arrangement (M = 5.93; SD = 1.07), and Usefulness (M = 6.69; SD = .61) subscales indicated an average degree of agreement above “somewhat agree” with usability statements. Furthermore, 13/14 (93%) caregivers reported a positive overall experience and agreed that the app was an acceptable method to monitor burn care. 12/14 (86%) caregivers reported that the app captured important clinical information. 53% preferred app-based burn care, 31% preferred both face-to-face and app-based care, and 15% preferred in-person only. Only 1 person preferred synchronous video-based care to asynchronous text-messaging. All study participants suggested improvements, with the most common being: (1) keeping user logged in, (2) time-stamping photos and messages, (3) consolidating text-messages and pictures, (4) adding push notifications and appointment reminders, and (5) tracking pain level.

**Conclusions:**

Mobile health technology may be leveraged to improve outpatient burn care. The results from this study (1) demonstrate caregiver experiences using a novel mHealth platform for outpatient pediatric burn care which showed high acceptability and usability and (2) provide systematic data for app optimization.

